# Evaluating glacier surges in Karakoram region using earth observation data

**DOI:** 10.1016/j.dib.2020.105394

**Published:** 2020-03-07

**Authors:** Ulfat Majeed, Irfan Rashid

**Affiliations:** Geoinformatics Program, School of Earth and Environmental Sciences, University of Kashmir, Hazratbal Srinagar, 190006, Jammu and Kashmir, India

**Keywords:** Glacier surges, Remote sensing, Glacier velocity, Karakoram

## Abstract

A glacier is said to be in a state of surge if it has abnormally high velocity and advances very rapidly (10–100 times faster than normal) in a short period of time (lasting few months to a few years). The glacier velocities can be easily assessed using feature-tracking techniques and image correlation algorithms. The applications of multi-source remotely sensed imagery from open source platforms for understanding glacier surges has been discussed in this paper. This paper draws insights for understanding glacier surges in Karakoram region using remote sensing data from two recently published papers (Rashid et al., 2020, 2018). The use of remote sensing data from United States Geological Survey (USGS) and Planet Labs for assessing glacier surface velocity, movement/advance of snout, supraglacial debris cover dynamics and evolution of ice-dammed lake has been discussed.

Specifications table

**Table:** 

Subject Area	Earth and Planetary Sciences
More specific subject area	Glaciology; Glacier surges
Type of data	Image and Vector, TIFF/IMG and SHP files
Data Acquisition	Raw satellite data products include moderate resolution Landsat 8 OLI datasets and high resolution data of Cubesat constellation. The datasets could be downloaded from USGS Earth Explorer (http://www.earthexplorer.usgs.gov/) and Planet Labs (http://www.planet.com) respectively. Processed data include velocity profiles computed from CIAS. Processed data also include snout changes, supraglacial moraine/debris cover dynamics and evolution of ice-dammed lake (as shapefiles) delineated in Arc Map 10.1.
Data format	Analyzed
Parameters for data collection	Satellite data devoid of clouds and shadows.
Description of data collection	Data acquired from the following web portals: http://www.earthexplorer.usgs.gov/; http://www.planet.com
Data source location	Name of the Glacier: Shisper glacier City/Town/Region: Hassanabad, Hunza Valley Country: Pakistan Latitude: 36.5°-36.48° N Longitude: 74.57°-74.61°E Elevation of Shisper Glacier: 2509–7234 m asl
Data accessibility	The datasets described in this study are available from the Mendeley repository and can be accessed at: https://data.mendeley.com/submissions/ees/edit/7v8t6xgh2c?submission_id=DIB_19212&token=d89d4afb-67e0-439c-8450-28f3bc643e4a
Glacier characterization	Glacier surface velocity changes; Snout advance; Supraglacial debris cover dynamics and Ice-dammed lake evolution
Related research article	Rashid, I., Majeed, U., Jan, A., Glasser, N.F. (2020) The January 2018 to September 2019 surge of Shisper Glacier, Pakistan, detected from remote sensing observations. Geomorphology, 351, 106,957. https://doi.org/10.1016/j.geomorph.2019.106957

## Value of the data

• The data/analysis can contribute towards better understanding of the glacier surges in the Karakorum and other glaciated regions across the world. While the temporal resolution of Landsat data is 16 days, the high resolution Planet images are available at daily time-step.
• Landsat data could be used to track velocity, debris cover distribution, folding and looping of supraglacial moraines twice a month provided the landscapes are not masked by cloud and/or snow.
• The Planet images could be important for tracking snout advance, ice-dammed lake development, dam-breach and infrastructure at risk.

## 1 Data description

This section discusses the remotely sensed datasets that were used to track the actively surging Shisper Glacier, Pakistan. These data are related to glacier surface velocity profiles, supraglacial moraine dynamics, snout advance, debris cover changes and evolution of glacial lake throughout the surge of Shisper glacier. The 16-day repeat coverage of Landsat 8 OLI (Spatial resolution: 30 m, PAN: 15 m) available since 2013 can help to precisely track changes on the surface of glacier. These changes could be related to ascertaining the looping and folding of supraglacial moraines, debris cover changes, and dynamics of supraglacial water bodies. The data pertaining to supraglacial moraines dynamics (moraines.rar) and debris cover changes (debris.rar) are provided in the Mendeley data repository. Surface velocity profiles, an important parameter to characterize glacier surge, for Shisper Glacier between January 2018 and September 2019 were quantified using the image correlation algorithm. The surface velocity profiles of Shisper Glacier at monthly time step have been provided in the Mendeley data repository (velocity.rar). The high resolution Planet images could be very important for tracking fine scale glacier changes, evolution of ice-dammed lakes and mapping flood affected areas downstream post Lake Outburst at a daily time-step. The snout changes (snout.rar) and evolution of ice-dammed lake (ice_dammed_lake.rar) has been delineated from Cubesat images provided by Planet labs.

## 2 Experimental design, materials and methods

The surface velocity profiles of Shisper Glacier on monthly time-step were quantified using Correlation Image Analysis System (CIAS) algorithm [1,3] from panchromatic band (spatial resolution: 15 m) of Landsat 8 OLI images. The CIAS algorithm requires coregistered images of the same area acquired in two different time periods to compute surface velocity profiles. This method has been widely used for assessing surface glacier velocity profiles across mountain regions [2,^4^,5].

The debris cover and moraines were delineated manually using on-screen digitization at 1:25,000 scale in GIS environment utilizing Landsat 8 OLI data (spatial resolution: 30 m) to capture the dynamics of these supraglacial features. Further, high resolution (3 m) Planet images were used to precisely track the snout advance of Shisper Glacier and development of ice-dammed lake at 1:2000 scale. Depending upon the availability of cloud/snow free satellite data and rationale of research, the data was generated at monthly or sub-annual time step.
